# Molecular optimization of rabies virus glycoprotein expression in *Pichia pastoris*

**DOI:** 10.1111/1751-7915.12350

**Published:** 2016-02-16

**Authors:** Safa Ben Azoun, Aicha Eya Belhaj, Rebecca Göngrich, Brigitte Gasser, Héla Kallel

**Affiliations:** ^1^ Laboratory of Molecular Microbiology, Vaccinology and Biotechnology DevelopmentBiofermentation UnitInstitut Pasteur de Tunis13, place Pasteur. BP. 74Tunis1002Tunisia; ^2^ Department of BiotechnologyBOKU ‐ University of Natural Resources and Life Sciences ViennaMuthgasse 18Vienna1190Austria

## Abstract

In this work, different approaches were investigated to enhance the expression rabies virus glycoprotein (RABV‐G) in the yeast *Pichia pastoris*; this membrane protein is responsible for the synthesis of rabies neutralizing antibodies. First, the impact of synonymous codon usage bias was examined and an optimized RABV‐G gene was synthesized. Nevertheless, data showed that the secretion of the optimized RABV‐G gene was not tremendously increased as compared with the non‐optimized one. In addition, similar levels of RABV‐G were obtained when α‐factor mating factor from *Saccharomyces cerevisiae* or the acid phosphatase PHO1 was used as a secretion signal. Therefore, sequence optimization and secretion signal were not the major bottlenecks for high‐level expression of RABV‐G in *P. pastoris*. Unfolded protein response (UPR) was induced in clones containing high copy number of RABV‐G expression cassette indicating that folding was the limiting step for RABV‐G secretion. To circumvent this limitation, co‐overexpression of five factors involved in oxidative protein folding was investigated. Among these factors only *PDI1*,*ERO1* and *GPX1* proved their benefit to enhance the expression. The highest expression level of RABV‐G reached 1230 ng ml^−1^. Competitive neutralizing assay confirmed that the recombinant protein was produced in the correct conformational form in this host.

## Introduction

The methylotrophic yeast *Pichia pastoris* (*Komagataella* sp.) has become a substantial workhorse for biotechnology, especially for heterologous protein production (Kurtzman, 2009; Ahmad *et al*., 2014). Using this system, a variety of proteins of different origins (human, animal, plant, fungal, bacterial and viral) has been produced with varying degrees of success (Sreekrishna *et al*., 1997; Leonardo *et al*., 2012).

However, not all recombinant proteins are efficiently secreted. High‐level expression in *P. pastoris* could face some potential bottlenecks, such as limitations in gene dosage, mRNA transcription, protein processing and folding in the endoplasmic reticulum (ER) (Agaphonov *et al*., 2002) and translocation which is depending on the secretion signal peptide (Koganesawa *et al*., 2001).

Genetic modification and molecular biotechnology proved to be most useful and effective tools for high‐level expression (Bai *et al*., 2011). Several genetic factors can be modified to enhance protein expression such as sequence optimization (Bai *et al*., 2011), gene copy number (Norden *et al*., 2011; Shen *et al*., 2012), promoter selection (Shen *et al*., 1998; Hohenblum *et al*., 2004), secretion signal (Koganesawa *et al*., 2001) and coexpression of folding‐assistant proteins (Li *et al*., 2010; Shen *et al*., 2012).

Previous investigations have demonstrated that the limiting steps are dependent on several factors such the protein to be expressed, the promoter and the host strain employed (Cereghino and Cregg, 2000; Li *et al*., 2007; Ashe and Bill, 2011). Generally, sequence optimization to adapt the codon usage of the gene of interest to the preferred host codon usage has been identified as one of the important factors influencing heterologous expression in *P. pastoris* (Cereghino and Cregg, 2000). For instance production of *Aspergillus niger* lipase (Yang and Liu, 2010), yeast multidrug resistance protein MDR1 (Bai *et al*., 2011) and *Streptomyces rimosus* GDS(L)‐lipase (Vujaklija *et al*., 2002) in *P. pastoris* were increased through codon optimization by 5.3‐fold, threefold and 22‐fold respectively. In addition, numerous studies have shown that gene dosage of the foreign protein has a high impact on recombinant protein production (Yu *et al*., 2009; Zhu *et al*., 2009). Yu *et al*. (2010) have demonstrated that increasing the gene copy number of lip2 from *Yarrowia lipolytica* enhanced the protein expression level by twofold. In many cases, increasing the target gene copy number dramatically enhances the production of foreign protein in *P. pastoris*. Although in some cases, such as human trypsinogen (Hohenblum *et al*., 2004) and Na‐ASPI (Inan *et al*., 2006) opposite results were obtained; increased gene dosage led to a reduction of the expression level. This was attributed to limitations in folding and secretion or a saturation of the secretory pathway. Secretory protein production usually requires the presence of a secretion signal sequence at the N‐terminus of the foreign protein to target it to the secretory pathway; different secretion signals are available to target protein secretion in *P. pastoris*, such as PHO1 secretion signal (Chang *et al*., 1986) and *Saccharomyces cerevisiae* α‐mating factor secretion signal (Payne *et al*., 1995). Each signal has its particular advantage, and there is no common rule which allows the identification of the most effective sequence (Hashimoto *et al*., 1998; Damasceno *et al*., 2012; Gasser *et al*., 2013).

During the journey of a protein through different cellular compartments, namely the ER, the Golgi apparatus, and finally, vesicular transport to the extracellular environment several post‐translational modifications occur (Vanz *et al*., 2014). However, not all recombinant proteins are efficiently secreted and ER retention during high‐level production can be a problem. In particular, aberrant folding properties of the target protein and/or high‐level production can lead to the accumulation of unfolded or even aggregated proteins in the ER (Inan *et al*., 2006; Hesketh *et al*., 2013) which can initiate the unfolded protein response (UPR) (Hohenblum *et al*., 2004; Whyteside *et al*., 2011; Zhu *et al*., 2011) and ER‐associated degradation (ERAD) (Whyteside *et al*., 2011; Vanz *et al*., 2012).

The ER contains several chaperones and foldases such as protein disulfide isomerase (PDI), the hsp70 member Kar2/BiP, calreticulin and calnexin, which are mainly involved in protein folding processes. Their co‐overexpression with the product gene has significantly improved the productivity of several secreted proteins (Inan *et al*., 2006; Damasceno *et al*., 2007; Li *et al*., 2010). Protein folding and ER stress have a severe impact on the redox state of the ER as well as on the cytosolic redox balance which can affect the process of folding its self as described in Delic *et al*. (2012). By altering the levels of redox active enzymes such as glutathione peroxidase (GPX) or the antioxidant transcription factor *YAP1*, the amount of a secreted heterologous protein was improved (Delic *et al*., 2012, 2014).

In this work, we report our efforts to enhance the heterologous production of rabies virus glycoprotein (RABV‐G) in *P. pastoris*. This protein which is exposed on the surface of rabies virus has been identified as the major antigen that induces protective immunity and thereby affords complete protection against rabies virus challenge (Cox *et al*., 1977). To identify the limiting steps in RABV‐G expression in this yeast we investigated the effect of different factors that can have a significant impact on heterologous gene expression; these factors are: gene optimization, secretion signal sequence, gene copy number and the coexpression of different proteins. In particular, we studied the effect of the coexpression of proteins which are involved in the oxidative protein folding process (*PDI1* and *ERO1*), those related to glutathione (*GPX1* and *GLR1*) and the transcriptional regulator factor *YAP1*, which responds to oxidative stress and was shown to be induced during methanol growth (Yano *et al*., 2009). *YAP1* activates the expression of the glutathione redox system including glutathione reductase (*GLR1*).

To explore the importance of codon bias and sequence optimization on the expression of RABV‐G in *P. pastoris*, the sequence of wild‐type (wt)‐RABV‐G gene was optimized for *P. pastoris* and the expression of optimized (opt)‐RABV‐G in the selected transformants was monitored by Western blot and enzyme linked immunosorbent assay (ELISA). To understand the effect of RABV‐G gene dosage, the copy number of the expression cassette inserted into host genome was determined by RT‐qPCR in the transformed clones and correlated with the expression level. Two secretion signals (α factor and PHO1) were also studied. Finally the effect of coexpression of five intracellular proteins (*PDI1*,* ERO1*,* GPX1*,* GLR1* and *YAP1*) on RABV‐G production level was investigated. The results of these explorations are presented in this study.

## Results

### Effect of gene optimization, signal sequence and gene dosage on RABV‐G production

Rabies virus glycoprotein is a type I membrane glycoprotein containing 505 amino acids in the native form, and it is the mediator of binding to cellular receptors and entry to host cells (Anilionis *et al*., 1981). It is composed of a cytoplasmatic domain, a transmembrane domain, and an ectodomain; the protein forms homotrimers and is anchored on the membrane envelope of the virion (Gaudin *et al*., 1992). RABV‐G has seven disulfide bonds and three potential N‐glycosylation sites (Gaudin *et al*., 1992; Walker and Kongsuwan, 1999). The important immunogenic property of RABV‐G makes it as an attractive alternative that can be used as a vaccine or as diagnostic antigen in ELISA for detecting anti‐glycoprotein antibodies in immunized host.

The expression of native RABV‐G gene in *P. pastoris* under the control of AOX1 promoter and using α‐factor as a secretion signal, has led a low expression level around 60 ng ml^−1^ (Ben Azoun *et al*., in press). It is worth to note that the expression of this membrane protein in this host was not reported so far in the literature.

In an effort to improve the expression level of RABV‐G by increasing translational efficiency, an optimized gene of RABV‐G (opt‐RABV‐G) supplied by GeneCust was used to transform *P. pastoris* cells. The sequence of wt‐RABV‐G was optimized with an appropriate algorithm that replaces rare codons by preferred ones in *P. pastoris* and optimizes other aspects of mRNA structure for optimal expression in this host. Some codons used in RABV‐G were converted to the high frequency codon preferences in *P. pastoris* summarized in Table S1. A total of 188 codons in the wt gene were changed to codon preferred by *P. pastoris*, 317 codons remained unchanged. To design the codon‐optimized gene, two restriction endonuclease sites were introduced at 5′ and 3′ ends of the coding sequence for an easy cloning.

To determine the effect of sequence optimization on the yield of RABV‐G two expression plasmids were constructed, the optimized sequence of RABV‐G gene was cloned in pPICZαA containing the α‐MF‐prepro secretion signal of *S. cerevisiae* and in pHIL‐S1 containing the acid phosphatase PHO1 signal sequence of *P. pastoris*. The expression plasmids containing the opt‐RABV‐G gene were linearized and transformed into the *P. pastoris* strains KM71H (pPICZαA) or GS115His^−^ (pHIL‐S1). The expression cassette was integrated into the *P*.*pastoris* genome at the 5′AOX1 locus via homologous recombination, giving rise to (His^+^; Mut^S^) and (His^+^; Mut^+^) phenotype in the transformants of KM71H and GS115 His^−^ respectively.

**Figure 1:**
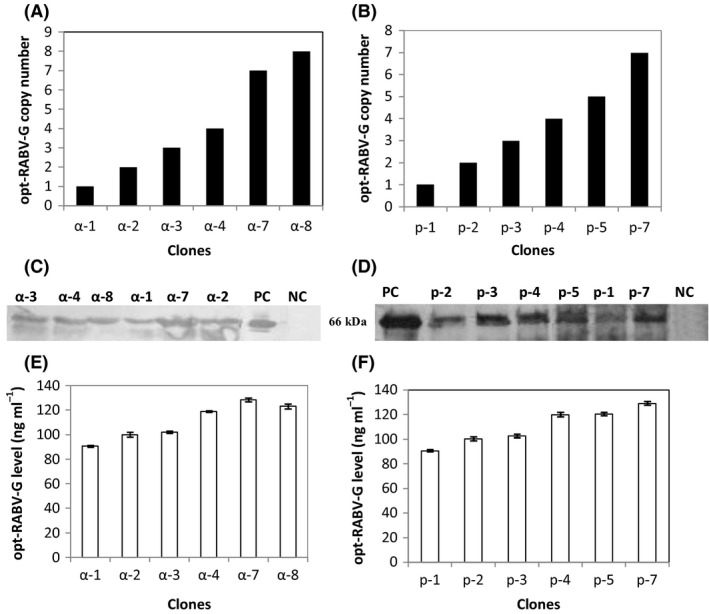
RABV‐G protein expression in the selected recombinant *Pichia pastoris* strains. Copy number of selected recombinant *P. pastoris* strains of (A) pPICZαA‐RABV‐G and (B) pHIL‐S1‐RABV‐G transformants. Western blot analysis of 15 μl of culture supernatants (concentrated 10‐fold by centricon) of (C) pPICZαA‐RABV‐G, and (D) pHIL‐S1‐RABV‐G transformants. PC (positive control): inactivated and purified rabies virus. NC (negative control): KM71H and GS115His^−^ strains transformed with empty pPICZαA and pHIL‐S1 vectors respectively. The amount of RABV‐G produced by the different clones with α‐factor (E) or PHO1 (F) signal sequence at 72 h of induction as determined by ELISA.

For each construction, we selected six recombinant strains and determined the copy number of the integrated expression cassette by real‐time q‐PCR (Fig. 1A). For clones transformed with pPICZαA‐opt‐RABV‐G, two clones named α‐8, harbouring eight copies of RABV‐G gene, and α‐7 containing seven copies were isolated. Clones bearing intermediate number of the expression cassette were also identified, and named α‐3 and α‐4. Clones with low copy were isolated, and designated as α‐1 and α‐2.

A similar approach was applied for clones transformed with pHIL‐S1‐opt‐RABV‐G and resulted in the isolation of clones containing different copies of the expression cassette. p‐7 clone contains seven copies, whereas p‐4 and p‐5 harbour intermediate copy number of the expression cassettes: four and five copies respectively. Clones with low copy number were isolated, and named p‐1, p‐2 and p‐3. These clones contain one copy, two copies and three copies of the expression cassette respectively (Fig. 1B).

The selected clones were cultivated in deep well plates, after induction of RABV‐G by methanol during 72 h; RABV‐G levels in culture medium were determined by Western blot using anti‐rabies polyclonal antibodies as shown in Fig. 1C and D. Only a protein band of 66 kDa corresponding to RABV‐G was clearly found in all culture supernatants of the recombinant strains. No band was detected in the transformed clones with empty vectors (pPICZαA and pHIL‐S1), suggesting that the RABV‐G protein was successfully secreted into the culture medium after methanol induction. The expression level of RABV‐G by the different clones was measured by ELISA. Figure 1E and F shows that RABV‐G level depends on the number of integrated expression cassette, but not on the type of signal secretion. Clones α‐7 and α‐8 bearing seven and eight copies, respectively, showed an expression level of 128.5 ng ml^−1^. For clones containing lower RABV‐G gene copies, we observed a correlation between the inserted copies of the expression cassette and the expression level; although no linear correlation was observed between these factors. Therefore, these data show that there is an optimal copy number of the inserted expression cassette beyond which the efficiency of folding is limited (Fig. 1E). p‐7 clone showed an expression level of 129 ng ml^−1^ which was similar to that obtained with α‐7 clone, harbouring the same copy number of the expression cassette but using PHO1 as a signal secretion.

These data indicate that sequence optimization had increased the expression level only by 2.1‐fold; nevertheless the use of different secretion signals did not show a marked impact on product yield. Therefore, these factors do not seem to be major bottlenecks for high‐level expression of RABV‐G. On the contrary, the improvement of the RABV‐G level seems to be due to the enhancement of RABV‐G transcription; the correlation between mRNA of RABV‐G transcript level, gene dosage and protein level is shown in Fig. S1.

The transcription level of the RABV‐G gene increased with the increase of gene dosage in both constructs (Fig. S1). The transcription levels of RABV‐G in clones containing equal copy numbers of α‐factor‐opt‐RABV‐G and PHO1‐opt‐RABV‐G were compared with respective clones harbouring one copy of each expression cassette. The transcription level of RABV‐G increased in clones containing two gene copies (α‐2 and p‐2) by 1.12‐fold when compared with the single copy clones (α‐1 and p‐1). For clones harbouring three and four copies of RABV‐G gene, namely α‐3, α‐4, p‐3 and p‐4, the enhancement of RABV‐G transcript level was higher and was in the range of 1.5‐fold for clones with three copies to 2.3 for clones with four gene copies.

Clones containing high copy number of each expression cassette showed a remarkable increase of mRNA level of RABV‐G gene. α‐7 and p‐7 clones which integrated seven copies of RABV‐G gene exhibited an enhancement factor of 4.7‐fold and 4.3‐fold respectively. Clone α‐8 which contains eight copies of the expression cassette reached the highest increase of RABV‐G transcript (5.5‐fold). These data show that there is a linear correlation between the mRNA level of the gene of interest and the expression level. The increase of gene dosage resulted in higher mRNA levels but did not significantly enhance the secreted RABV‐G protein level. This can be due to depletion of precursors and energy (Baumann *et al*., 2011) or to an accumulation of the protein in the cell caused by limitations in folding and/or secretion. These data also indicate that mRNA levels of RABV‐G gene were not the limiting factor for high‐level expression of RABV‐G, thus we thought that RABV‐G could be retained in the cell.

### Determination of intracellular RABV‐G protein in different recombinant clones

**Figure 2:**
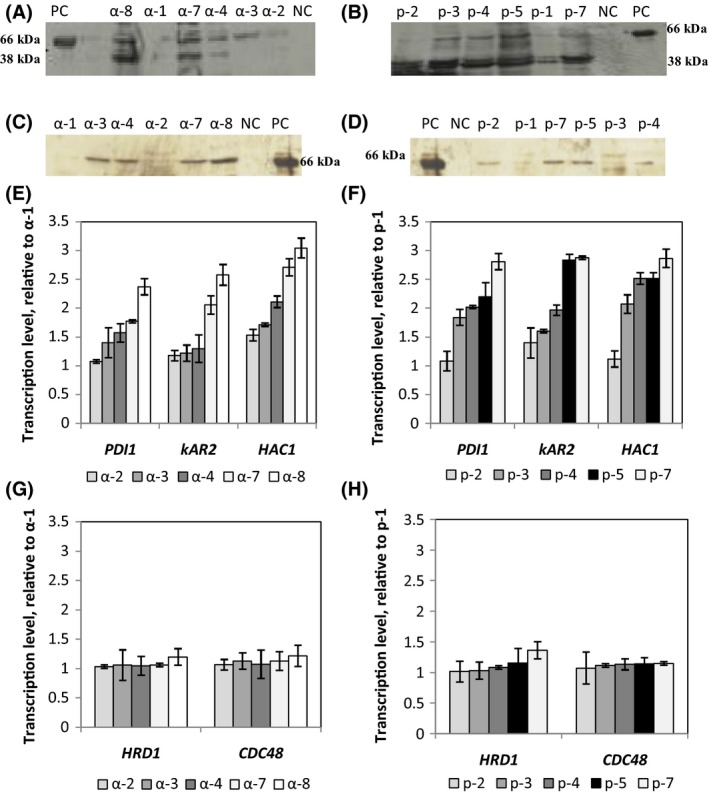
Soluble intracellular RABV‐G protein accumulation in clones with (A) α‐factor and (B) clones with PHO1 signal sequence. Membrane‐associated RABV‐G protein level in clones with (C) α‐factor and (D) in clones with PHO1 signal secretion. Transcription levels of selected UPR‐related genes (*PDI1*,*KAR2*,*HAC1*) in clones with (E) α‐factor and (F) PHO1 as a signal secretion. Transcription levels of ERAD‐related genes (*HRD1* and *CDC48*) in the different *Pichia pastoris* recombinant strains with α‐factor (G) and (H) PHO1 signal secretion. Gene transcript levels were normalized relative to α‐1 or p‐1 strains containing single copy of RABV‐G gene. α‐2, α‐3, α‐4, α‐7 and α‐8 correspond to selected clones of recombinant *P. pastoris* strains harbouring different copies of RABV‐G gene and where RABV‐G secretion was driven by the α‐factor. p‐2, p‐3, p‐4, p‐5 and p‐7: selected clones where RABV‐G was directed by PHO1 signal secretion and containing different copies of RABV‐G gene.

To check if RABV‐G protein was retained within the cell, cell extracts were divided into soluble and membrane‐associated fractions (including the secretory organelles) according to Hohenblum *et al*. (2004) and analysed by Western blot (Fig. 2). The intensity of bands obtained by different clones was more pronounced in the soluble fraction than in the membrane‐associated fraction for clones utilizing either α‐factor or PHO1 as a secretion signal. Figure 2 shows that the gene copy number impacts the number and the intensity of the intracellular product bands. High copy strains such as α‐8 and α‐7 showed more intense bands than intermediate copy strains (α‐4 and α‐3). By contrast, for low copy strains (α‐1 and α‐2), minor level of RABV‐G was seen in the soluble fraction (Fig. 2). In addition, for high copy strains (as α‐8 and α‐7), a band with a molecular weight lower than 66 kDa was observed. This band could be due to degradation of the protein of interest. For clones where the secretion of RABV‐G protein was directed by PHO1 signal, the accumulation of full length RABV‐G protein in the soluble fraction was lower than that seen for α‐factor clones (Fig. 2). Nevertheless, the degradation of RABV‐G protein was more prominent even for the low copy strains. However for α‐factor clones, the proportion of RABV‐G retained in the membrane‐associated fraction was more prominent than that obtained for PHO1‐strains. This effect was prominent for the high copy strains (Fig. 2C and D).

### Transcription study of key genes involved in UPR and ERAD pathway

To determine if the retention of RABV‐G inside the cell was due to folding limitations in the ER, the transcription levels of three typical UPR signal proteins (*HAC1*,* PDI1* and *KAR2*) were determined. In addition, the transcript levels of two key proteins involved in ERAD pathway (*HRD1* and *CDC48*) were measured, to determine if the UPR response had triggered an ERAD response.

The transcription levels of *HAC1* and the two chaperones located in the ER, *KAR2* and *PDI1* determined in all clones after methanol induction for 72 h are displayed in Fig. 2E and F. As compared with the strain containing one copy of RABV‐G gene, the transcription level of *HAC1* was 2.7‐fold and 2.8‐fold higher in α‐7 and p‐7, respectively, but lower in the low and medium copy number strains. On the other side *HAC1* transcript level elevated to threefold in the α‐8 strain which contains eight copies of the RABV‐G gene.

Therefore, increasing the RABV‐G copy number resulted in an expected increase in the *HAC1* mRNA level. These results suggest that RABV‐G over expression had induced the UPR in all strains. The transcription level of the other two UPR related genes confirmed this hypothesis. *PDI1* and *KAR2* transcripts showed a similar trend, with an increase of 1.7‐fold in α‐7 and 2.8‐fold in p‐7 for *PDI1* mRNA level, and 2.05‐fold in α‐7 and 2.8‐fold in p‐7 for *KAR2* transcript (Fig. 2E and F).

To determine the destiny of unfolded RABV‐G, the transcription level of two key genes involved in ERAD pathway were determined in clones with either the α‐factor or PHO1 secretion signal. Figure 2G and H shows the relative transcription levels of *HRD1* and *CDC48* in the different strains. No significant difference in transcription of these two genes in all clones was seen as compared with α‐1 or p‐1; this demonstrates that the ERAD pathway was not activated.

These data clearly suggest a folding limitation of the expressed protein. Therefore, we attempted to coexpress five proteins to improve the folding of RABV‐G protein. These proteins are (i) *PDI1* and *ERO1* which are the main players of the oxidative protein folding machinery in the ER; (ii) Glutathione‐related genes such as *GLR1* which is responsible for converting oxidized glutathione to reduced glutathione and *GPX1* involved in the detoxification of reactive oxygen species (ROS) at the expense of reduced glutathione, and (iii) Yap1which is the transcription factor of the oxidative stress response.

### Coexpression of the oxidative protein folding (PDI1 and ERO1)

Protein disulfide isomerase (*PDI1*) is an ER‐resident foldase which plays a crucial role in the formation, isomerization and reduction of disulfide bonds. During disulfide bond formation, *PDI1* accepts electrons from cysteine residues in nascent proteins, leading to the reduction of *PDI1* (Delic *et al*., 2012). ER oxido‐reduction (*ERO1*), on the other hand, is an enzyme which interplays with *PDI1* during this process and transfers the electrons to molecular oxygen or other electron acceptors to restore the oxidized form of *PDI1* (Tu *et al*., 2000).

Plasmids overexpressing *PDI1*or *ERO1* under control of glyceraldehyde‐3‐phosphate dehydrogenase (GAP) promoter were transformed by electroporation into α‐7/KM71H and p‐7/GS115^−^ strains that contained seven copies of RABV‐G gene.

Six clones from each construction were selected and their copy number was estimated by real‐time q‐PCR (Table S2). α‐7 clones containing low, medium and high copy numbers of *PDI1* gene, namely α‐7/P1, α‐7/P3 and α‐7/P6 were selected. p‐7/P1, p‐7/P6 and p‐7/P3 bearing different copy numbers of *PDI1* were also isolated. In addition, α‐7 and p‐7 clones harbouring different copy numbers of *ERO1* were selected. The production of RABV‐G by the selected clones was studied and compared with α‐7 and p‐7 strains which contain only seven copies of RABV‐G gene. The expression level was monitored by Western blot and ELISA of culture supernatants after 72 h of methanol induction.

**Figure 3:**
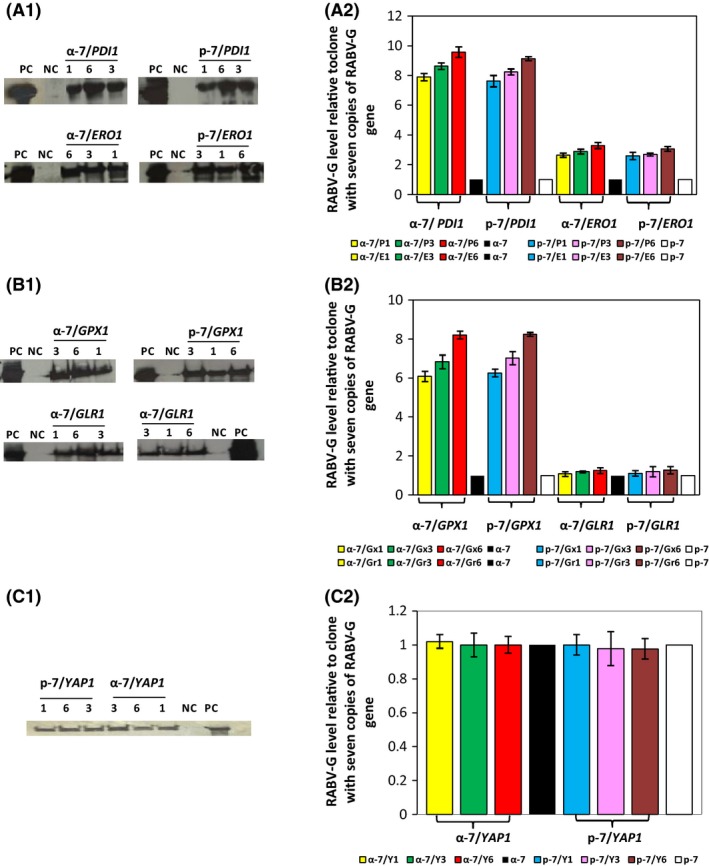
Effect of coexpression of five factors involved in oxidative protein folding on the expression level of RABV‐G. Western blot analysis of 15 μl of culture supernatants (concentrated 10‐fold by centricon) of yeast clones with α‐factor or PHO1 sequence leader to direct RABV‐G protein secretion coexpressing (A‐1), *PDI1* or *ERO1* genes, (B‐1) *GPX1* or *GLR1* and (C‐1) *YAP1* genes. PC (positive control): inactivated and purified rabies virus. NC (negative control): KM71H and GS115His^−^ transformed with an empty pPICZαA and pHIL‐S1 respectively. The numbers 1, 3 and 6 correspond to copy number of different genes contained in each clone. The amount of RABV‐G coexpressed with (A‐2) *PDI1* or *ERO1*, (B‐2) *GPX1* or *GLR1* and (C‐2) *YAP1* produced by the different clones with α‐factor or PHO1 as a signal sequence at 72 h of methanol induction as determined by ELISA. Clone abbreviations are explained in Tables S2, S3 and S4.

Western blot analysis of culture supernatants of the selected recombinant strains expressing *PDI1*or *ERO1* shows the presence of a single band at the expected size of RABV‐G (Fig. 3A‐1). The intensity of the band depends on the chaperone coexpressed. Coexpression of *PDI1* dramatically improved the level of secreted RABV‐G, independent of the secretion signal used; this effect increased with higher gene copy number of *PDI1*. Insertion of six copies of *PDI1* resulted in 9.6‐fold enhancement of secreted RABV‐G when compared with α‐7 and p‐7 strains. Insertion of three or one copy of *PDI1* increased the production level 8.6‐fold and 7.9‐fold respectively (Fig. 3A‐2).

*ERO1* coexpression improved RABV‐G expression in all clones, as seen in Western blot analysis of culture supernatants. However, when compared with *PDI1* expression, the effect was lower. Here, we observe an enhancement factor around three independent of the *ERO1* gene copy number (Fig. 3A‐1 and A2).

These results clearly demonstrate that *PDI1* overexpression led to a significantly higher level of RABV‐G when compared with *ERO1* strains.

### Coexpression of glutathione‐related genes (GPX1 and GLR1)

Glutathione reductase is a key enzyme in the conversion of oxidized GSSG to its reduced form GSH and is crucial for the maintenance of the cellular glutathione redox potential (Toledano *et al*., 2013). *GPX1*, on the other hand, is an enzyme involved in the detoxification of ROS in particular H_2_O_2_, at the expense of reduced glutathione, thereby generating GSSG (Toledano *et al*., 2013). The effect of these two enzymes on the RABV‐G production was evaluated, and the selection of α‐7 and p‐7 clones harbouring low, medium and high copy number of *GPX1* and *GLR1* genes was performed (Table S3).

The expression of the RABV‐G protein by the selected clones was analysed by Western blot and ELISA (Fig. 3B‐1 and B2). We showed that *GPX1*coexpression had a positive effect on the expression level of RABV‐G protein. A single protein band with the molecular weight of 66 kDa was revealed in all culture supernatants; its intensity increased with *GPX1* gene copy number (Fig. 3B‐1). This result was also confirmed by ELISA (Fig. 3B‐2); the insertion of six copies of *GPX1* gene in α‐7 and p‐7 strains enhanced the expression of RABV‐G protein by 8.2‐fold. The insertion of one or three copies of *GPX1* gene improved the expression level by six‐ and 6.8‐fold respectively. Coexpression of *GLR1* with RABV‐G had only a minor effect on the expression level of RABV‐G protein (Fig. 3B‐1 and B‐2). Gene dosage of *GLR1* does not seem to have a significant impact on the expression level for α‐7 and p‐7 strains, as *GLR1* coexpression enhanced RABV‐G production by 1.1‐ to 1.28‐fold for all clones. These data indicate that the coexpression of *GPX1* has a large impact on RABV‐G when compared with *GLR1*.

### Coexpression of the transcription factor in stress response (YAP1)

The transcription factor *YAP1* is a major oxidative stress regulator. During growth on methanol, *YAP1* activates the expression of the glutathione redox system and upregulates *GLR1* (Yano *et al*., 2009). The impact of the coexpression of this protein on RABV‐G production was evaluated in strains with low, medium and high copy number of *YAP1*, named as α‐7/Y1, α‐7/Y3, α‐7/Y6 and p‐7/Y1, p‐7/Y3, p‐7/Y6. The expression of RABV‐G was compared with α‐7 and p‐7 containing seven copies of RABV‐G gene (Table S4).

Overexpression of *YAP1* had no effect on the level of RABV‐G when compared with their respective controls (α‐7 and p‐7), as determined by Western bolt and ELISA (Fig. 3C‐1 and C‐2). RABV‐G expression level reached after 72 h of methanol induction remained unchanged for all clones.

### Competition of neutralization activity of rabies‐immune serum by RFFIT

**Figure 4:**
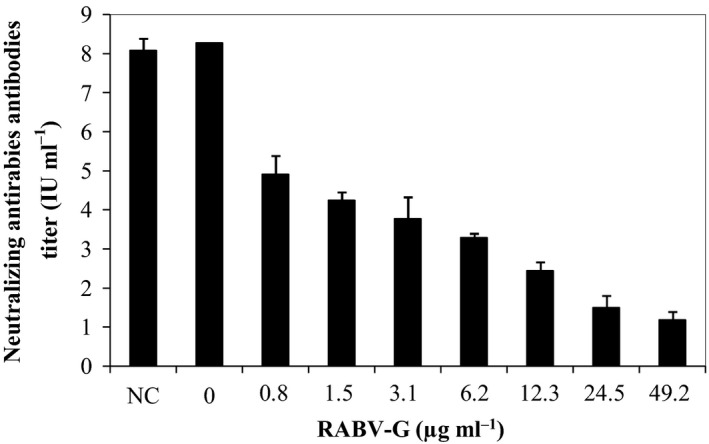
Competition of the neutralizing activity of the rabies‐immune serum by recombinant RABV‐G. The neutralizing titres (IU ml^−1^) were determined in the presence of varying amounts of RABV‐G expressed by α‐7/P6 recombinant clone, NC (negative control): culture supernatant of KM71H strain transformed with empty pPICZαA vector.

To prove that the RABV‐G expressed by clone α‐7/P6 was secreted in its native form and can compete with rabies virus to react with anti‐rabies neutralizing serum and to block cell infection, the RFFIT test was used with the modifications introduced by Li *et al*. (2010). The neutralizing activity of rabies‐immune serum was evaluated in the presence of different levels of recombinant RABV‐G varying from 0.8 to 49 μg ml^−1^. As shown in Fig. 4, the neutralizing titre of the rabies‐immune serum was significantly reduced in the presence of RABV‐G compared with that seen in the absence of recombinant RABV‐G. These results suggest that the RABV‐G produced by *P. pastoris* was recognized by rabies virus neutralizing antibodies.

## Discussion

High‐level expression of a given protein in a heterologous system can be affected by several factors; a practical solution is to identify major bottlenecks which are in general, host‐ and product‐dependent. Codon optimization, signal sequence and gene dosage are the main factors studied to improve the expression of a target protein. Several examples have been mentioned in literature that demonstrate that the use of an optimized sequence, the increase of gene copy number and the choice of leader sequence can significantly enhance protein productivity (Vujaklija *et al*., 2002; Zhao *et al*., 2009; Shen *et al*., 2012).

In this work, we first studied the effect of gene optimization on the expression of RABV‐G in *P. pastoris*. We showed that the expression of opt‐RABV‐G led to approximately 2.1‐fold increase in RABV‐G production compared with wt‐RABV‐G (S. Ben Azoun, unpublished). These data indicate that sequence optimization was not a critical parameter for RABV‐G expression in *P. pastoris*, as gene optimization sequence did not result in a tremendous increase of the expression level.

Numerous studies have demonstrated that the choice of secretion signal had a profound impact on the expression level of heterologous proteins (Ide *et al*., 2007; Ghosalkar *et al*., 2008). It controls the entry of proteins into the oxidative protein folding compartments such the ER in eukaryotes and the periplasm in prokaryotes (Killian *et al*., 1990; Von Heijne, 1990; Rapoport, 1992). For *P. pastoris*, few secretion leader sequences are known and applied for the secretion of heterologous proteins (Damasceno *et al*., 2012; Gasser *et al*., 2013). The most widely used secretion signal is the *S. cerevisiae* α‐mating factor leader sequence, whereas the *P. pastoris* acid phosphatase PHO1 signal sequence has been used in few cases (Heimo *et al*., 1997; Romero *et al*., 1997). The *S. cerevisiae* SUC2 gene signal sequence was used occasionally (Paifer *et al*., 1994). It was reported that the expression level of hIFN‐α2b in *P. pastoris* was higher with α‐factor signal sequence than with its native signal sequence (Ghosalkar *et al*., 2008). Expression of ocanase in *P. pastoris* under the direction of the PHO1 secretion signal was less effective than α‐factor (Zhao *et al*., 2009). While Koganesawa *et al*. (2001) demonstrated that the silkworm lysozyme expression using α‐factor leader was so unstable that it could be easily attacked by proteases, they suggested that the expression level and the stability of secreted heterologous protein were greatly affected by the selection of the appropriate secretion signal sequence.

PHO1 and α‐factor secretion leader were evaluated in this work for the secretory expression of RABV‐G in *P. pastoris*. We found that the expression level of secreted RABV‐G was similar in clones containing equal copies of the product gene from both constructs. However, intracellular RABV‐G retention profile was different; a band with a molecular weight lower than 66 kDa was observed. This band could be due to degradation of the protein of interest; this band was more important in clones where the secretion of RABV‐G protein was directed by PHO1 signal. These clones are derived from the strain *P. pastoris* GS115, they have the phenotype Mut^+^. Hence, they have faster cellular machinery on methanol compared with KM71H recombinant clones which are Mut^s^ (clones with α‐factor sequence leader).

The optimization of the gene copy number is another factor that can be modulated to improve the expression level in *P. pastoris*. Several studies have shown that this parameter is critical for heterologous protein expression in *P. pastoris* (Shen *et al*., 2012; Yang *et al*., 2013). We demonstrated that increasing RABV‐G copy number resulted in an increased RABV‐G transcript level as well as secreted RABV‐G level. This positive correlation between the copy number and the expression was only observed in clones bearing less than eight copies of RABV‐G gene, despite the high transcriptional rate observed in this clone. This can be due to the saturation of the folding capacity in this high copy number clone, and to the activation of UPR mechanism as described for secreted heterologous proteins in *S. cerevisiae* (Kauffman *et al*., 2002) or in *P. pastoris* (Inan *et al*., 2006).

The retention of RABV‐G in the *P. pastoris* strains was investigated, we have found that RABV‐G protein was partially accumulated in intracellular compartments; this accumulation increased with the gene copy number, implying that the secretory protein pathway might be saturated in high copy strains.

One can assume that the product is retained in the ER due to limitations in folding and/or disulfide bond formation and the set up of ER stress as a consequence. This triggers the activation UPR pathway which aims at reducing ER stress conditions by induction of genes involved in protein folding (Hoseki *et al*., 2010; Kohno, 2010). To test whether the UPR was induced, the transcript levels of three key genes (*HAC1*,* PDI1*,* KAR2*) involved in UPR pathway were analysed. Seventy‐two hours after induction of RABV‐G expression lead to a significant increase of *HAC1*,* PDI1* and *KAR2* transcripts and an accumulation of intracellular RABV‐G (Fig. 2). These data clearly indicate that UPR was induced upon induction of RABV‐G expression. Often the UPR is followed by ERAD (Lünsdorf *et al*., 2011; Vanz *et al*., 2012; Lin *et al*., 2013). The ERAD pathway has been investigated through the monitoring of the transcripts of two key genes (*HRD1*and *CDC48*). There was no significant difference between the recombinant strains and the negative control indicating that ERAD was not triggered in RABV‐G expressing *P. pastoris* (Fig. 2).

Protein folding in the ER is a critical step, both, in mammals and yeasts and is a prerequisite for secretion (Helenius *et al*., 1992; Inan *et al*., 2006). The newly synthesized heterologous protein enters the lumen of the ER; it encounters a change in the redox environment that ultimately promotes the formation of intra‐chain and/or inter‐chain disulfide bonds (Inan *et al*., 2006). Chaperone proteins play a critical role in protein folding in the ER and efficient processing is necessary to obtain high levels of proteins (Ngiam *et al*., 2000).

Coexpression of the oxidative protein folding factors *PDI1* or *ERO1* has been successful in increasing the amount of some heterologous proteins in *P. pastoris* (Lodi *et al*., 2005; Baumann *et al*., 2011; Vad *et al*., 2005; Inan *et al*., 2012) although in some cases this approach was not effective to improve heterologous protein expression (Damasceno *et al*., 2007).

In the current study, coexpression of the two folding factors *PDI1* or *ERO1* remarkably increased the expression level of RABV‐G by 9.5‐fold and 3.3‐fold, respectively, in the high copy of RABV‐G gene strains (Fig. 3). This indicates that restriction in folding or misfolding of RABV‐G are the major bottlenecks for optimal expression of this protein. Recently, Delic *et al*. (2012) proved that protein folding and ER stress have a severe impact on the cytosolic redox balance which may be a major factor during folding.

In *P. pastoris*, several genes of gluthatione redox system, such as the gluthatione reductase gene (*GLR1*), the *GPX1*, are induced by the transcriptional regulator *YAP1* which responds to oxidative stress (Yano *et al*., 2009).

In this study, we coexpressed the glutathione‐related genes *GPX1* and *GLR1* and the transcription factor involved in stress response *YAP1*. It has been reported by Delic *et al*. (2012) that coexpression of *GPX1* has improved the level of trypsinogen 1.6‐fold in glucose‐driven protein production conditions compared with *GLR1* which failed to increase the production of this protein in *P. pastoris*. *GPX1* overexpression correlated with more oxidizing redox conditions in the ER, as did *PDI1* overexpression but not *ERO1* or *GLR1* overexpression (Delic *et al*., 2012). On the other hand, *YAP1* overexpression increased trypsinogen secretion by twofold and re‐established cytosolic redox state to the state of glucose‐grown wt strains (Delic *et al*., 2014).

The coexpression of *GPX1*,* GLR1* or *YAP1* genes in high RABV‐G copy strains yielded different results. The highest relative improvement was observed with clones that overexpressed *GPX1*, which showed 8.2‐fold increase of RABV‐G expression level, whereas the coexpression of *GLR1* slightly increased the RABV‐G expression (1.2‐fold). These results clearly show that cell engineering towards conditions previously shown to be beneficial for protein secretion by enhancing ER oxidation in glucose‐grown *P. pastoris* is also improving production of secreted product in methanol grown cells.

Surprisingly, the coexpression of YAP1 has failed to enhance RABV‐G expression; conversely the yield of RABV‐G protein was slightly decreased in the clone α‐7/Y6 (Fig. 3). Hence, overexpression of YAP1 gene is not required to enhance RABV‐G expression, the available YAP1 level allows the cells to manage with oxidative stress. This result is in line with those reported by Yano *et al*. (2009) who identified the *P. pastoris* YAP1 homologue and showed the involvement of this transcription factor in the detoxification of formaldehyde and ROS in *P. pastoris* cells grown on methanol. Delic *et al*. (2014) also showed that YAP1 is involved in physiological detoxification of ROS formed upon oxidative folding in the ER. YAP1 is required to activate the antioxidant enzymes, which are quenching ROS. Cells with significantly lowered YAP1 level react to increased secretory folding load with accumulation of ROS and strong flocculation.

Therefore, YAP1 level is already high in methanol grown cells (Yano *et al*., 2009); this might explain why the increase of YAP1 gene copies does not elicit any further antioxidant response.

However, it still remains to be analysed in future if the reduction of the cytosolic redox state upon production of secretory proteins and ER stress observed in glucose conditions (Delic *et al*., 2012, 2014) is also occurring in methanol grown *P. pastoris*, or if the constant high YAP1 level on methanol prevent or diminish this response.

The RABV‐G protein produced by *P. pastoris* was able to significantly reduce the neutralizing activity of the human immune rabies serum, thus demonstrating that the recombinant protein was produced in the correct conformational form.

In conclusion, in the current study we investigated different approaches to enhance RABV‐G expression in *P. pastoris*. The data obtained showed that using an optimized gene sequence, different signal sequence or increasing gene copy number were not the major bottlenecks to improve the amount of recombinant RABV‐G. Coexpression of oxidative folding proteins such as *PDI1* or *ERO1* and the glutathione‐related genes (*GPX1* or *GLR1*) improved the secretion of RABV‐G by 9.5‐fold, 3.3‐fold, 8.2‐fold and 1.2‐fold respectively. The secreted RABV‐G protein was also able to react with neutralizing antirabies serum, demonstrating therefore a correct folding of the recombinant protein. Overall, these results demonstrate through combined engineering of the expression construct and the cellular oxidative protein folding machinery, *P. pastoris* can produce sufficient amounts of functional recombinant RABV‐G which can be used as diagnostic antigen for detecting rabies virus neutralizing antibodies in immunized hosts or as a vaccine.

## Experimental procedures

### Strains, plasmids and media

*Pichia pastoris* KM71H and GS115His^−^ (Invitrogen, Carlsbad, CA, USA) were used as host strains for protein expression. *Escherichia coli* DHB10 (Invitrogen) was used for the propagation of recombinant vectors.

The plasmids pPICZαA with alpha factor secretion signal (Invitrogen) and pHIL‐S1 with PHO1 signal sequence were used for cloning and expression of opt‐RABV‐G protein (GenBank accession number KT878717). The plasmid Ppuzzle (Delic *et al*., 2012) was used for cloning and expression of five folding‐assisting factors: *PDI1* (GenBank accession number EU_805807.1), *ERO1* (GenBank accession number XM_002489600.1), *GPX1* (GenBank accession number AB_472088.1), *GLR1* (GenBank accession number AB_472087.1) and *YAP1* (GenBank accession number AB_472084.1). Gene expression was under control of the glycolytic glyceraldehyde‐3‐phosphate dehydrogenase promoter P_GAP_.

Yeast peptone dextrose (YPD) agar (2% peptone, 1% yeast extract, 2% glucose and 20 g l^−1^ agar) with zeocin (100 mg ml^−1^) was used to select the positive clones which contain the recombinant plasmid pPICZαA‐opt‐RABV‐G.

RDB (Regeneration Dextrose Medium + His) agar lacking histidine (1 M sorbitol, 1% glucose, 1.34% yeast nitrogen base, 4 × 10^−5^% biotin, 0.005% of L‐glutamic acid, L‐methionine, L‐lysine, L‐leucine and L‐isoleucine, and 20 g l^−1^ agar) was employed to select the His‐prototrophic clones transformed with the recombinant plasmid pHIL‐S1‐opt‐RABV‐G.

BMGY (Buffered Glycerol‐complex Medium) (1 mM potassium phosphate pH 6, 2% peptone, 1% yeast extract, 1.34% yeast nitrogen base, 1% glycerol and 4 × 10^−5^% biotin) was used for cell growth before the induction of recombinant clones of *P. pastoris* (KM71H, GS115His^−^) strains.

BMMY (Buffered Methanol‐complex medium) (1 mM potassium phosphate pH 6, 2% peptone, 1% yeast extract, 1.34% yeast nitrogen base, 1% methanol and 4 × 10^−5^% biotin) containing methanol as a carbon source was used to induce the expression of RABV‐G protein.

### Construction of expression vectors containing the optimized rabies virus glycoprotein sequence

Codon‐opt‐RABV‐G was obtained from GeneCust (Dudelange, Luxembourg). The opt‐RABV‐G lacking its native signal peptide sequence was cloned in two plasmids containing two different secretion signal sequences: pPICZαA with *S. cerevisiae* α‐mating factor as secretion signal and pHIL‐S1 with *P. pastoris* PHO1 as signal sequence.

The opt‐RABV‐G was amplified by PCR from the recombinant plasmid PUC 19 (GeneCust) using two different primer pairs listed in Table S5. αG‐F/αG‐R primers were used to clone RABV‐G gene into pPICZαA, the second pair pG‐F/pG‐R was employed for the cloning of RABV‐G gene into pHIL‐S1.

After amplification, PCR products were purified and ligated into pPICZαA or pHIL‐S1 and transformed into *E. coli*; positive clones from the ligation reactions were analysed by restriction digestion and sequencing of the insert fragment.

### Construction of expression vectors containing PDI1, ERO1, GPX1, GLR1 and YAP1sequences

*P. pastoris* genes *PDI1*,* ERO1*,* GPX1*,* GLR1* and *YAP1* were isolated from the recombinant pPuzzle vectors (Delic *et al*., 2012) which contain a zeocin selection marker. The genes were cloned into a pPuzzle vector under control of the GAP promoter and with KanMX as selection marker. The vectors were integrated into the 5′AOX1 region of *P. pastoris* recombinant clones harbouring seven copies of RABV‐G gene, after linearization of the respective sequences. After transformation by electroporation, positive transformants of *P. pastoris* were selected on YPD plates with zeocin and G418 or YPD plates with G418.

### Expression of recombinant Opt‐RABV‐G protein in deep 24‐well cell culture plates

Recombinant *P. pastoris* clones were grown in YPD agar plates or RDB agar plates at 30°C. For expression studies, 2 ml of BMGY in culture plate (Dominique Dutscher, Brumath, France) were grown overnight at 30°C and 250 r.p.m. After 14–16 h, optical density was measured at 600 nm, and cells were resuspended in 2 ml of fresh BMMY medium to an initial OD_600_ of 1. Cultures were performed at 250 r.p.m., 30°C up to 72 h with methanol added to 1% every 24 h. Aliquots were taken every 24 h, cells were pelleted; supernatants and cells were stored at −20°C for further analysis.

### Lysis of cells and detection of proteins

Cell pellets (the equivalent of 1 ml at an OD_600_ of ~50) were washed twice in phosphate buffered saline, then resuspended in 500 μl of yeast breaking buffer (50 mM sodium phosphate, 1 mM PMSF (Phenylmethylsulfonyl floride), 1 mM EDTA (Ethylenediaminetetraacetic acid) and 5% (v/v) glycerol), as described by Shen *et al*. (2012) and mixed with an equal volume of acid‐washed glass beads (Sigma Aldrich, St Louis, MO, USA). The yeast cell wall was broken by vortexing 10 times for 1 min with 1 min chilling on ice. The lysate was centrifuged at 16 000 *g* for 20 min at 4°C. The supernatants containing the cytosolic proteins were collected. The pellet containing the membrane proteins was further treated with 400 μl yeast breaking buffer plus 2% (w/v) SDS. After centrifugation at 4000 *g* for 5 min at 4°C, the supernatants containing the membrane proteins were collected and analysed by Western blot.

### Western blot analysis

Secreted proteins, soluble proteins and membrane proteins were analysed on a 12% SDS‐polyacrylamide gel using Bio‐Rad cell system. Proteins were transferred to a nitrocellulose membrane (GE Healthcare, Uppsala, Sweden). The membrane was incubated overnight at 4°C and then incubated with horse anti‐rabies virus polyclonal antibody for 1 h. After washing, the membrane was incubated with HRP‐conjugated polyclonal anti‐horse IgG (antibodies online, Germany). The membrane was finally incubated for two minutes with ECL solution (GE Healthcare).

### Enzyme‐linked immunosorbent assay test

An indirect ELISA was performed to quantify of RABV‐G expressed by the different clones. About 100 μl per well of either the sample or the standard (inactivated and purified rabies virus) were incubated for 2 h at 37°C. Thereafter, monoclonal antibody anti‐glycoprotein TW1 (NIBSC, Hertfordshire, UK) was added to the wells and incubated for 1 h at 37°C. Finally, anti‐human antibody coupled to peroxidase (Sigma Aldrich) were added and incubated 30 min at 37°C. After tetramethylbenzidine addition, the reaction intensity was measured at 450 nm. OD values higher than 0.150 were considered as positive.

### Competitive neutralization assay

The neutralizing titre of rabies‐immune serum was determined by RFFIT according to the standard methodology (Smith *et al*., 1973; Li *et al*., 2010). To determine whether RABV‐G expressed in *P. pastoris* reacted with neutralizing antibodies present in the human immune rabies serum, serial dilutions of recombinant RABV‐G were mixed with 50 μl rabies human immune serum (European Pharmacopeia, Strasbourg, France) and incubated at 37°C for 1 h. The serum and RABV‐G mixtures were then incubated at 37°C for 1 h with a constant dose of challenge rabies virus that causes infection in 80% of BHK‐21 cells. BHK‐21 cells were then added to the samples and incubated for 20 h at 37°C, 5% CO_2_. BHK‐21cells were fixed in 80% acetone stained with fluorescein‐labelled anti‐rabies nucleocapsid immunoglobulins (Sanofi Diagnostic Pasteur, Marnes la Coquette, France) to detect the presence of non‐neutralized virus (fluorescent foci).

### RNA extraction

For transcript quantification, frozen cells were resuspended in Trizol reagent (Invitrogen) and disrupted with glass beads in FastPrep^™^ cell homogenizer (Thermo Scientific, Waltham, MA, USA). Total RNA was then extracted using the RNeasy Kit from Qiagen (Hilden, Germany), following the manufacturer's instructions. RNA was tested in 1% agarose gel, and was quantified by measuring OD 230/260/280 using a NanoDrop (Thermo Scientific).

### Quantitative real‐time PCR to determine transgene copy number and gene transcriptional level

Individual reactions were carried out in 10 μl containing 5 μl Maxima^™^ SYBR^®^ Green qPCR Master Mix (Roche, Mannheim, Germany), 0.25 μl fw‐ and rev‐primers (listed in Table S5) and 4 ng template genomic DNA or 2.5 μg cDNA. RT‐qPCR was performed using a thermal cycler (Bio‐Rad, Hercules, CA, USA). PCR conditions were as followed: pre‐incubation at 95°C for 10 min, the thermal cycler was programmed to perform 45 cycles of: 15 s at 95°C; 20 s at 60°C; 15 s at 72°C. A melting curve was carried out to ensure that only a specific amplification product was obtained. RT‐qPCR was run in duplicate with biological replicates (independent experiments) to allow for the statistical confidence in differential gene expression.

### Data analysis

The relative gene expression was calculated for each sample; biological replicates (independent experiments) were conducted for all studies. As amplification efficiencies of the target and reference genes were not the same, Pfaffl method (Pfaffl, 2005) was chosen for the relative quantification of RT‐qPCR results.

## Conflict of Interest

The authors declare that they have no conflict of interest.

## Supporting information


**Fig. S1.** (A‐1) Correlation between mRNA level of RABV‐G gene, gene copy number and RABV‐G protein level (α‐RABV‐G and p‐RABV‐G correspond to the expression level of RABV‐G in *P. pastoris* clones expressing RABV‐G protein with α‐factor or PHO1 as signal sequence; α‐RNA, p‐RNA correspond to mRNA levels of RABV‐G gene in *P. pastoris* clones, where RABV‐G secretion was directed by α‐factor or PHO1 signal sequence).
**Table S1.** Codon preference in *P. pastoris* and comparison of codon usage frequency (%) in the wild type and the optimized synthetic RABV‐G genes.
**Table S2.** Copy number of helper factor genes (*PDI1*,* ERO1*) of *P. pastoris* clones coexpressed with RABV‐G.
**Table S3.** Copy number of helper factor genes (*GPX1*,* GLR*1) of *P. pastoris* clones coexpressing RABV‐G gene and containing seven copies of the expression cassette.
**Table S4.** Copy number of the helper factor *YAP1*gene of *P. pastoris* clones coexpressing RABV‐G sequence.
**Table S5.** List of oligonucleotides used in this study. Underlined sequences indicate restriction sites used for gene construction.
