# Supplementary material for: Molecular optimization of rabies virus glycoprotein expression in Pichia pastoris
Source: Microb Biotechnol. 2016 Feb 16;9(3):355–68. doi: 10.1111/1751-7915.12350 (PMC4835572; doi:10.1111/1751-7915.12350)
Supplement: Supplementary file 1 — Fig. S1. (A‐1) Correlation between mRNA level of RABV‐G gene, gene copy number and RABV‐G protein level (α‐RABV‐G and p‐RABV‐G correspond to the expression level of RABV‐G in P. pastoris clones expressing RABV‐G protein with α‐factor or PHO1 as signal sequence; α‐RNA, p‐RNA correspond to mRNA levels of RABV‐G gene in P. pastoris clones, where RABV‐G secretion was directed by α‐factor or PHO1 signal sequence). Table S1. Codon preference in P. pastoris and comparison of codon usage frequency (%) in the wild type and the optimized synthetic RABV‐G genes. Table S2. Copy number of helper factor genes (PDI1, ERO1) of P. pastoris clones coexpressed with RABV‐G. Table S3. Copy number of helper factor genes (GPX1, GLR1) of P. pastoris clones coexpressing RABV‐G gene and containing seven copies of the expression cassette. Table S4. Copy number of the helper factor YAP1gene of P. pastoris clones coexpressing RABV‐G sequence. Table S5. List of oligonucleotides used in this study. Underlined sequences indicate restriction sites used for gene construction. [file MBT2-9-355-s001.docx]

**Supporting information**

**Molecular optimization of rabies virus glycoprotein expression in *Pichia pastoris***

Safa Ben Azoun^1^, Aicha Eya Belhaj^1^, Rebecca Göngrich^2^, Brigitte Gasser^2^, Héla Kallel^1^

^1^Laboratory of Molecular Microbiology, Vaccinology and Biotechnology Development, Biofermentation Unit.Institut Pasteur de Tunis. 13, place Pasteur. BP. 74.1002 Tunis. Tunisia

^2^ Department of Biotechnology, BOKU - University of Natural Resources and Life Sciences Vienna, Muthgasse 18, 1190 Vienna, Austria

*Corresponding author: Héla Kallel. Laboratory of Molecular Microbiology, Vaccinology and Biotechnology Development, Biofermentation Unit. Institut Pasteur de Tunis. 13, place Pasteur. BP 74.1002 Belvédère.Tunis. Tunisia

Phone number: + 216 71 783 022

Fax number: + 216 71 791 833

e-mail: hela.kallel@pasteur.rns.tn

**Table S1.** Codon preference in *P.pastoris* and comparison of codon usage frequency (%) in the wild type and the optimized synthetic RABV-G genes

| **Codon** | ***P.pastoris***  **Kazusa database** | **Wt-RABV-G gene** | **Opt-RABV-G gene** |
| --- | --- | --- | --- |
| **Lys (K)**  AAA  AAG | 46.9%  53.1 % | 42.8%  57.1% | 3.6%  96.4% |
| **Phe (F)**  TTC  TTT | 46.1%  53.9 % | 66.6 %  33 .3 % | 16.6%  83.3% |
| **Pro (P)**  CCT  CCA  CCC  CCG | 34.8 %  41.6%  15%  8.6 % | 31.1%  24.1%  27.6 %  17.2% | 0 %  100%  0 %  0% |
| **Ile (I)**  ATT  ATA  ATC | 50.5 %  18 %  31.5 % | 25 %  37.5 %  37.5 % | 95.8 %  0%  4.16 % |
| **Tyr (Y)**  TAC  TAT | 53.1 %  46.9 % | 58.8 %  41.2 % | 100 %  0% |
| **Thr (T)**  ACG  ACC  ACT  ACA | 10.6%  25.6%  39.5%  24.3% | 9.1%  45.45%  21.21%  24.24% | 0%  15.1%  84.8%  0.00% |
| **Asp (D)**  GAC  GAT | 42.1%  58% | 60.7%  39.2% | 13.1%  86.9% |
| **Leu (L)**  CTT  CTC  TTG  CTG  CTA  TTA | 16.5%  7.9%  32.7%  15.5%  11.1%  16.2% | 20.8%  18.7%  22.9%  14.6%  6.2%  16.7% | 0%  0%  91.8%  8.16%  0%  0% |
| **Gly (G)**  GGT  GGA  GGG  GGC | 43.6%  32.7%  9.9%  13.9% | 20%  25%  37.5%  17.5% | 100%  0%  0%  0% |
| **Trp (W)**  TGG | 100% | 100% | 100% |
| **Ser (S)**  AGC  TCA  TCC  TCT  AGT  TCG | 9.1%  18.2%  19.7%  29.2%  15%  8.9% | 11.6%  27.9%  16.3%  20.9%  13.9%  9.3% | 0%  0%  16.3%  83.7%  0%  0% |
| **His (H)**  CAT  CAC | 56.5%  43.5% | 33.3%  66.7% | 100%  0% |
| **Cys (C)**  TGC  TGT | 36.4%  63.6% | 60%  40% | 0%  100% |
| **Asn (N)**  AAC  AAT | 51.5%  48.5% | 47.6%  52.4% | 100%  0% |
| **Val (V)**  GTA  GTG  GTT  GTC | 15.5%  19.2%  42%  23.3% | 27.1%  27.1%  30.8%  27.1% | 0%  0%  92.1%  7.9% |
| **Glu (E)**  GAG  GAA | 43.7%  56.3% | 61.3%  38.7% | 3.2%  96.7% |
| **Met (M)**  ATG | 100% | 100% | 100% |
| **Ala (A)**  GCC  GCT  GCA  GCG | 25.7%  44.8%  23.4%  6.1% | 31.6%  15.8%  42.1%  10.5% | 5.3%  94.7%  0%  0 % |
| **Arg (R)**  AGA  CGC  CGA  AGG  CGT  CGG | 48%  5.3%  10%  15.8%  16.5%  4.5% | 65.4%  7.7%  7.7%  11.5%  3.8%  3.8% | 92.30%  0%  0%  0%  7.7%  0% |
| **Gln (Q)**  CAA  CAG | 60.9%  39.1% | 71.4%  28.6% | 100%  0% |

**Table S2.** Copy number of helper factor genes (*PDI1*, *ERO1*) of *P.pastoris*  clones coexpressed with RABV-G. α-7 and p-7 clones correspond to recombinant clones harboring 7 copies of RABV-G gene and where the secretion was directed by α-factor (α-7) or PHO1 (p-7) signal sequence.

| Strain | RABV-G  copy number | *PDI1* copy number | *ERO1* copy number |
| --- | --- | --- | --- |
| α-7 | 7 | Endogenous 1 | Endogenous 1 |
| p-7 | 7 | Endogenous 1 | Endogenous 1 |
| α-7/P1 | 7 | 1 | Endogenous 1 |
| α-7/P3 | 7 | 3 | Endogenous 1 |
| α-7/P6 | 7 | 6 | Endogenous 1 |
| p-7/P1 | 7 | 1 | Endogenous 1 |
| p-7/P3 | 7 | 3 | Endogenous 1 |
| p-7/P6 | 7 | 6 | Endogenous 1 |
| α-7/E1 | 7 | Endogenous 1 | 1 |
| α-7/E3 | 7 | Endogenous 1 | 3 |
| α-7/E6 | 7 | Endogenous 1 | 6 |
| p-7/E1 | 7 | Endogenous 1 | 1 |
| p-7/E3 | 7 | Endogenous 1 | 3 |
| p-7/E6 | 7 | Endogenous 1 | 6 |

**Table S3.** Copy number of helper factor genes (*GPX1*, *GLR*1) of *P.pastoris* clones co-expressing RABV-G gene and containing 7 copies of the expression cassette. α-7 and p-7 clones correspond to recombinant clones where RABV-G secretion was directed by α-factor (α-7) or PHO1 (p-7) signal sequence.

| Strain | RABV-G  copy number | *GPX1* copy number | *GLR1* copy number |
| --- | --- | --- | --- |
| α-7 | 7 | Endogenous 1 | Endogenous 1 |
| p-7 | 7 | Endogenous 1 | Endogenous 1 |
| α-7/Gx1 | 7 | 1 | Endogenous 1 |
| α-7/Gx3 | 7 | 3 | Endogenous 1 |
| α-7/Gx6 | 7 | 6 | Endogenous 1 |
| p-7/Gx1 | 7 | 1 | Endogenous 1 |
| p-7/Gx3 | 7 | 3 | Endogenous 1 |
| p-7/Gx6 | 7 | 6 | Endogenous 1 |
| α-7/Gr1 | 7 | Endogenous 1 | 1 |
| α-7/Gr3 | 7 | Endogenous 1 | 3 |
| α-7/Gr6 | 7 | Endogenous 1 | 6 |
| p-7/Gr1 | 7 | Endogenous 1 | 1 |
| p-7/Gr3 | 7 | Endogenous 1 | 3 |
| p-7/Gr6 | 7 | Endogenous 1 | 6 |

**Table S4.** Copy number of the helper factor *YAP1*gene of *P.pastoris* clones coexpressing RABV-G sequence. α-7 and p-7 clones correspond to recombinant clones harboring 7 copies of RABV-G gene and where the secretion was directed by α-factor (α-7) or PHO1 (p-7) signal sequence.

| Strain | RABV-G  copy number | *YAP1* copy number |
| --- | --- | --- |
| α-7 | 7 | Endogenous 1 |
| p-7 | 7 | Endogenous 1 |
| α-7/Y1 | 7 | 1 |
| α-7/Y3 | 7 | 3 |
| α-7/Y6 | 7 | 6 |
| p-7/Y1 | 7 | 1 |
| p-7/Y3 | 7 | 3 |
| p-7/Y6 | 7 | 6 |

**Table S5.** List of oligonucleotides used in this study. Underlined sequences indicate restriction sites used for gene construction

| Primers | Sequence (5’-3’) | Restriction enzyme |
| --- | --- | --- |
| αG-F | TACTGAATTCAAGTTTCCAATTTACACTATTCCA | EcoRI |
| αG-R | AGTAGGTACCTTACAAACCAGTTTCACCACCAGACTTGTA | KpnI |
| pG-F | TACTCTCGAGAAGTTTCCAATTTACACTATTCCA | XhoI |
| pG-R | AGTAGAATTCTTACAAACCAGTTTCACCACCAGACTTGTA | EcoRI |
| Actin F | CCTGAGGCTTTGTTCCACCCATCT |  |
| Actin R | GGAACATAGTAGTACCACCGGACATAACGA |  |
| AOXTT F | GACTGGTTCCAATTGACAAGC |  |
| AOXTT R | GCAAATGGCATTCTGACATCC |  |
| Opt-RABV-G F | TCCATTGGCTGATCCATCTACTG |  |
| Opt-RABV-G R | CCAGTTTGGCAAACCCAGATC |  |
| GPX1F | CTATTGTCGGATTTCCCTGTAACCAGTTT |  |
| GPX1R | TCCGAACCGTTGACATCAATCTTTTT |  |
| GLR1F | CAAGGGGACAATGAGAAAGTGGTT |  |
| GLR1R | CAGGTCCTCGTTGGTGAAGA |  |
| PDI1 F | GGAAAGGCCCACGATGAAGTTGTC |  |
| PDI1 R | GCATCCTCATCATTGGCGTAAAGAGTAG |  |
| KAR2 F | TGGTCGTAGTGCCATTTGCT |  |
| KAR2 R | ATTTCCACACGACCCGACTT |  |
| HAC1 F | GCGGCCCATGCTTCCAGAGAG |  |
| HAC1 R | CGGTACCACCTAAGGCTTCCAACC |  |
| YAP1 F | CAGGCCAACTACCGTCACCAACTTCTA |  |
| YAP1 R | AGCCATCCACAGACTCATCAAAT |  |
| ERO1 F | GTTGGAAAAGCCGCATATAAACAAAACA |  |
| ERO1 R | CAGCTTGGGCAAAGTCCTGTAAGAGTTC |  |
| HRD1 F | TGGCCAACTTCGTGATAGCA |  |
| HRD1 R | CCCAAGACCGCTCAAAAACA |  |
| CDC48 F | GCACAGAAAATGGCCCTGAA |  |
| CDC48 R | TCACCTCGGAACAACTGCAA |  |

F: Forward R: Reverse

**Figure S1**

**Figure S1**. (a-1) Correlation between mRNA level of RABV-G gene, gene copy number and RABV-G protein level (α-RABV-G and p-RABV-G correspond to the expression level of RABV-G in *P.pastoris* clones expressing RABV-G protein with α-factor or PHO1 as signal sequence; α-RNA, p-RNA correspond to mRNA levels of RABV-G gene in *P.pastoris* clones where RABV-G secretion was directed by α-factor or PHO1 signal sequence). The relative transcription level of RABV-G gene in six recombinant *P.pastoris* clones with α- factor (a-2) and PHO1 signal sequence (a-3) tested after 72h of methanol induction
